# Epigenetic Biomarkers of Renal Cell Carcinoma for Liquid Biopsy Tests

**DOI:** 10.3390/ijms22168846

**Published:** 2021-08-17

**Authors:** Raimonda Kubiliute, Sonata Jarmalaite

**Affiliations:** 1 Life Sciences Center, Institute of Biosciences, Vilnius University, Saulėtekio Av. 7, 10257 Vilnius, Lithuania; raimonda.kubiliute@gmc.vu.lt; 2 National Cancer Institute, 08660 Vilnius, Lithuania

**Keywords:** renal cell carcinoma, epigenetic alterations, DNA methylation, miRNAs, lncRNAs, non-invasive detection, liquid biopsy

## Abstract

Renal cell carcinomas (RCC) account for 2–3% of the global cancer burden and are characterized by the highest mortality rate among all genitourinary cancers. However, excluding conventional imagining approaches, there are no reliable diagnostic and prognostic tools available for clinical use at present. Liquid biopsies, such as urine, serum, and plasma, contain a significant amount of tumor-derived nucleic acids, which may serve as non-invasive biomarkers that are particularly useful for early cancer detection, follow-up, and personalization of treatment. Changes in epigenetic phenomena, such as DNA methylation level, expression of microRNAs (miRNAs), and long noncoding RNAs (lncRNAs), are observed early during cancer development and are easily detectable in biofluids when morphological changes are still undetermined by conventional diagnostic tools. Here, we reviewed recent advances made in the development of liquid biopsy-derived DNA methylation-, miRNAs- and lncRNAs-based biomarkers for RCC, with an emphasis on the performance characteristics. In the last two decades, a mass of circulating epigenetic biomarkers of RCC were suggested, however, most of the studies done thus far analyzed biomarkers selected from the literature, used relatively miniature, local, and heterogeneous cohorts, and suffered from a lack of sufficient validations. In summary, for improved translation into the clinical setting, there is considerable demand for the validation of the existing pool of RCC biomarkers and the discovery of novel ones with better performance and clinical utility.

## 1. Introduction

Based on its incidence in both sexes, kidney (or renal) cancer takes fourteenth place worldwide and is among the top ten most common cancers in males (according to IARC, https://gco.iarc.fr/, accessed on 7 July 2021). Renal cell carcinoma (RCC), originating from the epithelium of the nephron tubules, is the most common type of kidney cancer, accounting for 90% of all cases, and is the most lethal cancer of the urinary system [1,2]. The three major subtypes of RCC are clear cell RCC (ccRCC), representing the most common and aggressive form (70–80%), papillary RCC (pRCC), accounting for 10–15%, and chromophobe RCC (chRCC), accounting for 5% of RCC; meanwhile, the remaining subtypes are very rare [3]. While the majority of patients will present with localized disease, 25–40% of those treated with curative intent will develop distant disease and 20–25% of patients will present with metastatic disease at diagnosis [4], which is, presumably, the source of the significant health burden of RCC. This is attributed to the characteristic lack of symptoms associated with primary RCC and, currently, the majority of patients are diagnosed incidentally due to the extensive use of radiology imaging for the investigation of various non-specific symptoms [5,6]. The possibility for diagnosing disease using liquid biopsy-based molecular biomarker tests, along with imaging, could not only enhance early diagnosis, but also facilitate patients’ follow-up and differentiation into low and high-risk progression groups.

The potential sources for disease biomarkers include tumor tissue (biopsy) and bodily fluids, such as urine, blood serum, or plasma. Recent studies analyzing multiregional and sequential tumor samples by genome-wide mutation analyses [7,8,9] suggested a high heterogeneity of ccRCC tumors, which is overlooked in the single biopsy studies, and even 73–75% of driver alterations were found to be subclonal [10]. Biopsies are less appropriate for patients’ follow-up due to hazardous and painful procedures. Thus, to date, “liquid biopsy” is emerging as a revolutionary tool in cancer care with some important advantages over tissue biopsy. First of all, intratumor heterogeneity may be captured better in body fluids, reflecting a wider spectrum of (epi)genetic alterations from various tumor foci and even micrometastatic spots. Most importantly, concerning its non-invasive (urine) or minimally invasive (blood) nature, liquid biopsies can be obtained frequently and, due to the ease of availability, repeatability, and comparability, allow for the detection of cancer at an early stage or the following of the real-time state of the malignant transformation and disease progression.

The recent study by Mitchell et al. [7] revealed that clonal expansion of ccRCC tumors is dilatory and a long period of time (up to 50 years) is required from the initial genetic alteration to clinical manifestation of a tumor. While hardly any histological change is evident in the corresponding histologically normal renal tissue of patients with renal tumors, epigenetic alterations will have already accumulated in such non-cancerous renal tissues [11], suggesting their suitability for early diagnosis of the disease. Epigenetic phenomena, particularly DNA methylation, microRNAs (miRNAs), and long noncoding RNAs (lncRNAs), can modulate gene expression and such changes are frequent and related to various clinical subgroups of RCC [12,13,14]. In addition, in comparison with genetic alterations, epigenetic changes are more pronounced and frequent in all RCC subtypes [15]. Moreover, epigenetic marks can be easily detected in the body fluids, such as urine or peripheral blood samples, by conventional and inexpensive qualitative or quantitative PCR methods. Thus, epigenetic alterations may serve as non-invasive biomarkers that could provide clinicians with rapid, objective, and accurate tools for the detection of and follow-up on renal tumors.

Despite their potential, no diagnostic and prognostic non-invasively detectable RCC-specific epigenetic biomarkers have reached the clinical setting yet; meanwhile, DNA methylation-based tests for other urological cancers (prostate and bladder) have been commercially available for a long period of time [16]. Navigation toward clinical utility is challenging and requires representative, large, and preferably multiregional patient series as well as sufficient validations. Here, we provide an overview of currently described potential DNA methylation-, miRNA-, and lncRNA-based urine and blood circulating biomarkers for kidney cancer detection and/or prognosis without detailing the technical issues thoroughly described elsewhere [17,18]. To provide a summary of the present knowledge, a systemic search using PubMed was performed (updated 7 July 2021). The literature search encompassed the terms “DNA methylation AND renal cell carcinoma AND urine/serum/plasma”, “miRNA AND renal cell carcinoma AND urine/serum/plasma” and “lncRNA AND renal cell carcinoma AND urine/serum/plasma”. In addition, references of the relevant publications were reviewed to include additional eligible research. It is worth mentioning that only manuscripts describing adult RCC cases were discussed, while investigations related to genetic syndromes, such as Wilms tumors, were excluded.

## 2. Biofluid DNA Methylation as the Biomarkers for Renal Cell Carcinoma

DNA methylation in mammalian cells is characterized by the addition of a methyl group (-CH3) at the carbon-5 position of cytosine residues in the context of CpG dinucleotides through the action of DNA methyltransferase (DNMTs) enzymes, forming 5-methylcytosine (5mC). It is the most widely studied epigenetic mechanism, responsible for various biological processes including the normal development of mammals, differentiation, and regulation of gene expression [19]. Promoter CpGs in normal cells generally remain unmethylated (hypomethylated) and are associated with active gene expression during differentiation. However, hypermethylation of the promoter CpGs is a common event in various cancer types, including kidney cancer, and is often associated with the silencing of tumor suppressor genes and downstream signaling pathways [19,20]. Alterations in DNA methylation occur early during cancer development and, in the case of ccRCC, are observable even in the precancerous stage [11,21] with increasing promoter hypermethylation frequencies in higher stage and grade tumors [22]. Aberrant DNA methylation is easily detectable in body fluids by conventional PCR-based methods. Considering the relatively infrequent number of somatic mutations and slow progress of clonal expansion until cancer diagnosis [7,23], DNA methylation could be precious clinical cancer biomarkers for the early diagnosis and prognosis of kidney cancer.

Despite the high potential of DNA methylation as the disease biomarker, only 12 studies shed light on efforts to analyze DNA methylation in liquid biopsy specimens as non-invasive biomarkers for RCC [24,25,26,27,28,29,30,31,32,33,34,35] (Table 1), encompassing 25 individual genes. Four (33%) of 12 studies discussed used urine as the source of methylated DNA, six studies (50%) reported on serum or plasma, and two (17%) described both urine and serum/plasma. Most of the biomarkers studied were classic tumor suppressor genes known to be involved or methylated in several human cancers [36], and only one study based their biomarker selection on gene expression microarrays data performed on renal cancer cell lines [27]. In addition, one study used high-throughput sequencing to detect RCC [35]. For the validation, the ruling methods were the bisulfite conversion-based MSP or qMSP with rare exceptions (Table 1). Only two studies by Outeiro-Pinho et al. [34] and Nuzzo et al. [35] performed internal validation with training and test sample sets, while others missed this step. In addition, 58% of the studies included ≤50 RCC patients and only two studies [32,34] used a homogenous study cohort composed of patients with ccRCC, while others involved heterogeneous groups of patients with various types of renal tumors [24,27,28,29,31,33,35] and, in some cases, the particular subtype was not specified [25,26,30].

Most studies compared circulating methylated DNA data with matched (or not) tissue samples [24,25,26,27,30,33,34], while no identical DNA methylation patterns between primary tumor and liquid biopsies were identified, and, as a rule, lower methylation frequencies or intensities in the body fluids were detected. The diagnostic sensitivity of various individual biomarkers varied from 6% to 83%, while the specificity was generally high and reached >90% for most of the biomarkers (Table 1). Interestingly, Hoque et al. [25] found higher methylation frequencies of *APC*, *ARF*, *GSTP1*, *P16*, *RARB2*, *RASSF1A,* and *TIMP3* in the urine samples when compared to the matched serum samples, while specificities were almost the same. However, too few samples (*n* = 17) were compared to conclude that urine was a more sensitive tool for cancer diagnosis.

In five studies [24,25,27,29,31], biomarker combinations were evaluated with the best performing combination of six biomarkers, namely *VHL*, *P16*, *P14*, *APC*, *RASSF1A*, and *TIMP3*, achieving 90% sensitivity and 100% specificity in the urine samples [24]. Skrypkina et al. [31] found a similar sensitivity (92%) for the panel of only three genes, *RASSF1A*, *FHIT*, and *APC*, in the plasma samples; however, the specificity was lower (87%).

The most innovative research on non-invasive RCC detection was performed by Nuzzo et al. [35], who used cell-free methylated DNA immunoprecipitation and high throughput sequencing (cf-MeDIP-seq) for highly sensitive detection of early-stage tumors. The investigators performed cf-MeDIP-seq on plasma cell-free DNA samples and identified differentially methylated regions (DMRs) between patients and control groups to build a classifier. The top 300 DMRs were selected, enabling accurate detection of all stages of RCC with an AUC = 0.99 and an AUC = 0.86 in the plasma and urine samples, respectively. Moreover, the created classifier strongly distinguished RCC from urothelial bladder cancer in the plasma samples with an AUC = 0.98. However, due to complexity, the translational potential of such a classifier to clinical practice is currently limited.

Seven studies in total revealed significant associations of circulating DNA methylation biomarkers with clinical-pathological variables [26,28,29,30,32,33,34]. Urakami et al. [26] found a higher methylation frequency of all genes in combination (*SFRP1*, *SFRP2*, *SFRP3*, *SFRP4*, *SFRP5*, *DKK3*, and *WIF1*) in higher grade and higher stage RCC. Not surprisingly, de Martino et al. [28] found higher *VHL* methylation in patients with ccRCC compared to other subtypes, but no associations were observed among other clinical-pathological variables. Houser et al. [29] described a higher methylation level of *APC* in patients with pT3 tumors when compared to pT1 stage RCC. Xin et al. [30] reported a positive association of *TCF21* methylation level, tumor stage, and Fuhrman grade as well as a clinical-stage. Lin et al. [32] correlated *PCDH17* methylation with higher tumor stage, grade, and lymph node metastasis. Jung et al. [33] found a positive correlation between *SHOX2* methylation and T, N, and M categories, histopathological grade, and lymphovascular invasion. Finally, Outeiro-Pinho et al. [34] observed higher urinary levels of methylated miRNA gene mir-30a in patients with an advanced pathological stage and those that recurred or developed metastasis during follow-up.

The independent prognostic value of circulating methylated DNA biomarkers was reported in only three studies discussed [32,33,34]. Lin et al. [32] defined *PCDH17* methylation as an independent factor for worse progression-free survival (PFS) and overall survival (OS) of patients with ccRCC; the adjusted (by sex, age, stage, grade, and lymph node metastasis) HRs were 4.0 and 3.9, respectively. Jung et al. [33] showed a significantly higher risk of death for patients with an increased blood plasma level of methylated *SHOX2* with an HR of 1.5, and the association remained significant even after adjustment to the tumor stage. Outeiro-Pinho et al. [34] described an association between higher levels of methylated urinary mir-30a and shorter metastasis-free survival and disease-specific survival (DSS); however, in the multivariable analysis, methylated mir-30a depicted an independent prognostic value for only DSS. No such associations were found, however, in the independent study cohort, which perfectly reflects the necessity of validating such results.

In summary, although 25 individual DNA methylation biomarkers for non-invasive detection and/or follow-up of patients with renal cancer were published, only 12 of them were investigated in an independent study or population. Among all biomarkers, only a few individual markers (*TCF21*, *LRRC3B*, and mir-30a) and multimarker panels (investigated by Battagi et al. [24], Hoque et al. [25], Urakami et al. [26], Houser et al. [29], and Skrypkina et al. [31]) showed sensitivities >70%, thereby making them potentially promising diagnostic biomarkers. However, these markers and panels either were studied in small heterogeneous populations [24,25,26,29,30,31] or the discriminating ability was plumped to a clinically insignificant level after validation in the larger cohort [34]. In addition, the majority of studies examined well-known tumor suppressor genes also known to be methylated in several cancer types, thus non-specific for RCC. In the future, next-generation sequencing-based hunting of biomarkers in biofluids seems to be the most promising tool for biomarker discovery.

## 3. Biofluid miRNAs as Biomarkers for Renal Cell Carcinoma

MicroRNAs (miRNAs) are a group of small, non-coding RNAs, 18–25 nucleotides in length, which regulate target gene expression by binding to the complementary 3’UTR of mRNA and inhibiting its translation to the protein or promoting degradation [37,38]. Accumulating pieces of evidence suggest the involvement of miRNAs in many processes related to cancer development and progression, including angiogenesis [39,40], cell proliferation [41,42], apoptosis [43,44], metastasis [45,46], invasion, as well as drug and radiation resistance [46,47,48]. The aberrant expression of miRNAs in renal cell carcinoma was observed by several independent studies, and evidence showed their involvement in RCC pathogenesis [49,50]. Moreover, miRNAs can be detected in various sources of biofluids, including serum, plasma, saliva, and urine [51]. The circulating miRNAs are protected from the endogenous RNase activity by binding with some proteins (e.g., Argonaute 2 protein) and lipoproteins [52,53] or due to the protection by secretory particles, such as apoptotic bodies, microvesicles, and exosomes [54,55], resulting in the remarkable stability of these molecules in biofluids. Indeed, circulating miRNAs are stable against degradation by RNase, pH changes, and freezing/thawing [56], thus may serve as non-invasive biomarkers. The profile of miRNAs expression is similar in men and women as well as in individuals characterized by different ages [57], which is possibly the main advantage miRNAs, as biomarkers, has over DNA methylation.

While compared to DNA methylation analysis, considerably more studies (43 discussed here), were completed concerning the topic of miRNAs as non-invasive biomarkers for RCC, including seven multicenter studies [58,59,60,61,62,63,64,65,66,67,68,69,70,71,72,73,74,75,76,77,78,79,80,81,82,83,84,85,86,87,88,89,90,91,92,93,94,95,96,97,98,99,100] (Table 2), encompassing >70 unique miRNAs. The vast majority (77%) of the studies (33 in total) used blood as the source of RNA, and serum was more common than plasma (used in 21 and 12 studies, respectively) despite the observation that the coagulation process may affect the spectrum of extracellular miRNAs in the blood, namely the platelet-derived ones [101]. Surprisingly, only 10 studies (23%) using urine samples for miRNA analysis in patients with RCC were reported. Seven studies specifically focused on the exosome- or microvesicles-derived miRNAs [72,74,78,88,89,96,99]. More than half of the studies based their biomarkers selection procedure on literature search, focusing on miRNA biomarkers studied in the kidney or in other cancer types. Biomarker selection in the remaining studies, on the other hand, was performed by the mining of specific databases (e.g., TCGA or Gene Expression Omnibus) or after initial miRNA screening by TaqMan Low-Density Arrays (TLDA), miRNA expression microarrays, and even the sequencing of liquid biopsy specimens [78,85,89,90] to select RCC-specific candidate biomarkers. In addition, most of the studies (89%) lacked an internal validation with training and validation sets or lacked the performance of internal validation approaches. Moreover, relatively few studies included >100 RCC patient samples in the validation step, and almost a third of studies investigated <50 of samples only.

Most of the studies (92%) primarily focused on the diagnostic objective of discrimination between patients with RCC and healthy or cancer-free controls. The study cohorts consisted either of patients with only clear cell RCC, or a heterogeneous group of patients, including papillary, chromophobe, or sarcomatoid RCC, as well as benign renal tumors (e.g., oncocytomas and angiomyolipomas), and only one study exclusively investigated serum samples obtained solely from pRCC patients [95]. Some studies compared circulating miRNA data with matched RCC and normal tissue [58,59,60,63,64,65,66,71,76,79,82,84,87,88,91,94], but the observed expression changes were not always concordant. For example, Zhao et al. [91] found the opposite regulation of miR-625-3p in the ccRCC tissue and serum samples, which was explained by the selective release of miRNAs from tumor cells.

Twenty studies reported clinically relevant (AUC ≥ 0.75) discriminating abilities of various miRNAs or their combinations, encompassing 33 distinct miRNAs in total (Table 2). The panels of miRNA were generally recommended to improve the accuracy of results and such panels were evaluated in the eight studies discussed. The highest diagnostic ability of such combinations was found by Liu et al. [90] for miR-508-3p and miR-885-5p, which had an AUC = 90 in both, with testing and validation sets of serum samples, and by Cochetti et al. [100] for miR-122, miR-1271, and miR-15b, which had an AUC = 96 in a very small set of urine samples. Yadav et al. [76] found an even better clinical value for only one miRNA, namely miR-1233, which had a superior AUC, equal to 0.97, and a sensitivity and specificity of 93% and 100%, respectively; however, only 30 ccRCC cases were included in this study. The requirement of validating such results in the independent and larger cohorts was perfectly conveyed in the multicenter study by Wulfken et al. [58], where the discriminating ability of the same miR-1233 reached only 0.67 and 0.59 of AUC in the testing and validation cohorts, respectively. Zhang et al. [72] also found a clinically useful AUC (0.82) for the exclusively exosomal miR-1233. It’s worth mentioning that these inconsistencies among the studies may come from different qRT-PCR analysis and normalization methods as well. For example, Sanders et al. [102] demonstrated that cel-miR-39, which was also used in the studies by Yadav et al. and Wulfken et al., was effective for the normalization of circulating miRNA in patients with urological malignancies, including RCC; meanwhile, RNU6, which was used by Zhang et al., was not a stable, normalization control [87].

Among urine-based diagnostic biomarkers, the study of Butz et al. [74] is the worthiest of mention. The authors reported acceptable discriminative abilities (AUC = 0.77–0.85) of various combinations of two exosomal miRNAs not only among ccRCC and healthy controls, but between healthy controls and small renal masses (SRM) and benign renal tumors (BRT) as well. Overall, miR-126-3p combined with miR-449a or miR-34b-5p could significantly distinguish ccRCC from healthy participants with an AUC of 0.84 and 0.79, respectively. The combination of miR-126-3p and miR-449a or miR-126-3p and miR-34b-5p was also able to distinguish SRM or BRT from healthy controls with an AUC of 0.89/0.79 and an AUC of 0.77/0.82, respectively. In addition, the authors found that after surgery, the expression of these miRNA returned to a level comparable with healthy control/status.

In many studies, changes of miRNA levels in the body fluid samples were observed after nephrectomy for treatment of RCC [60,63,70,72,73,74,75,79,80,84,86,87,88,92,94,96,97], suggesting the possibility of such miRNAs in follow-up monitoring of patients with RCC. In addition, the nine studies, encompassing 12 separate miRNAs, specifically miR-378 [70], miR-144-3p [79], miR-210 [83,88], miR-1233 [83], miR-22 [84] miR-122-5p, miR-206 [85], miR-15a [86], miR-508-3p, miR-885-5p [90], has-miR-328-3p [93], has-miR1293, and has-miR-301-3p [96] reported the association between the level of particular miRNA and clinical-pathological parameters, including tumor size, tumor stage, Fuhrman grade, necrosis, and cancer progression or metastasis. In more detail, deregulated expression of miR-15a were correlated with tumor size; miR-378, miR-144-3p, miR-22, miR-206, miR-210, miR-508-3p, and miR-885-5p were related with advanced tumor stage; miR-1233, miR-122-5p, miR-206, miR-210, miR-15a, miR-508-3p, and miR-885-5p with advanced tumor Fuhrman grade; miR-15a with tumor necrosis; and miR-210, miR-1233, miR-22, miR-508-3p, has-miR-328-3p, has-miR1293, and has-miR-301-3p with tumor progression or metastasis.

The prognostic value of circulating miRNAs was reported in seven of the studies [67,70,77,78,83,85,93]; however, only four studies [67,78,83,85] demonstrated that miRNAs expression could independently predict the survival of patients with RCC. The elevated expression level of miR-221 was associated with shorter OS and augmented the predictive ability of the tumor stage, Fuhrman grade, and patient age (≥60 years) from HR: 4.7 to HR: 10.7 in the multivariate model [67]. However, Du et al. [78] related the lower expression of miR-let-7i-5p, hsa-miR-26a-1-3p, and hsa-miR-615-3p with shorter OS, and miR-let-7i-5p remained significantly associated with patient survival with an HR of 0.57 after adjusting for the MSKCC score (the most common scoring system used for prognostication). Dias et al. [83] demonstrated the association between the increased plasma level of miR-210 and miR-1233 and cancer-specific survival (CSS), while the multivariate Cox regression model, using tumor TNM stage, Fuhrman grade, age (>60 years), and gender as co-variants, demonstrated a higher risk of disease-specific death in patients characterized by a simultaneously higher level of miR-210, miR-221, and miR-1233, with an HR: 3.02. Heinemann et al. [85] showed an association between decreased levels of miR-122-5p and miR-206 and patients’ CSS, PFS as well as OS, and a Cox regression model revealed miR-206 as an independent biomarker for PFS (HR: 3.5 while adjusted according to tumor TNM stage and grade). While circulating levels of miR-378, miR-150, and miR-328-3p were related to patients’ disease-free survival, disease-specific survival, and overall survival, respectively, they lacked evidence as an independent prognostic factor in RCC [70,77,93]. It is worth mentioning that all miRNAs stated as independent prognostic biomarkers lacked internal validation, and only Heinemann et al. [85] included >100 samples, while other authors investigated≤65 samples.

MiR-210 was the most widely studied circulating miRNA in the case of RCC, and was discussed in the nine studies reviewed herein. Despite significantly different experimental conditions (different miRNA isolation kits, PCR reagents, and quantification strategies), all investigators found increased circulating miR-210 levels in patients with RCC as compared to healthy controls. It is well known that miR-210 is expressed in response to hypoxia, mainly through HIF-1α, a key player of renal carcinogenesis [103]. However, it is worth mentioning that the upregulation of circulating miR-210 was also found in various other malignancies, [104] as well as non-cancerous conditions [105,106], and further validations, with suitable controls, are mandatory. MiR-1233 was also an actively studied circulating miRNA found upregulated in RCC in four studies; however, its functions have remained unresolved thus far. MiR-378 was an extensively studied circulating miRNA, however, the findings were quite divergent. Comparing RCC patients and healthy individuals, three studies reported an increase in miR-378 levels, while one study demonstrated a decrease. MiR-378 may act as both a tumor suppressor (inhibit cell proliferation and invasion) [107] or onco-miR (promote cell proliferation, migration, and invasion) [108] depending on the particular tissue. Other miRNAs, including miR-141, miR-150, miR-21, miR-34a, miR-508-3p, miR-15a, and miR-210-3p, were also studied in more than one report, while most of the miRNAs were investigated in a single study only, thus validation is urgently required.

The lack of knowledge about the biological function and role of particular miRNAs in renal carcinogenesis is another major obstacle to their use in clinical settings. Thirteen studies attempted to determine the molecular function of particular miRNAs [59,65,69,71,74,82,83,84,88,89,91,94,97] in renal carcinoma cells. Ten studies, encompassing nine distinct miRNAs, specifically miR-508-3p [59], miR-187 [65], miR-210 [71,88], miR-429 [82], miR-22 [84], miR-30c-5p [89], miR-625-3p [91], miR-765 [94], and miR-483-5p [98], significantly related their deregulation with either increased cell proliferation, migration, invasion, viability, and reduced apoptosis in vivo and, in some cases, with tumor growth in vivo [65,89,94]. In several of these studies, possible targets of the given miRNAs were investigated. Zhao et al. [65] revealed B7H3 as one of the miR-187 targets. Knockdown of B7H3 inhibited cell proliferation and migration, while downregulation of miR-187 reversed these processes [65]. Li et al. [84] showed that miR-22 inhibited cell proliferation and invasion by targeting epidermal growth factor receptor member ERBB3. Song et al. [89] reported the heat shock protein HSPA5 as the miR-30c-5p target. As the increased level of HSPA5 enhanced cell viability and colony formation ability, the downregulated miR-30c-5p contributed to tumor progression. Xiao et al. [94] discloses that miR-765 restrained cell proliferation, migration, and invasion by targeting endoplasmic reticulum protein PLP2, whose own expression is related with epithelial-mesenchymal transition (EMT) and G2M checkpoint, and, thus, with more aggressive tumors. Moreover, Wang et al. [97] showed the ability of miR-483-5p to inhibit cell migration and invasion through increased expression of E-cadherin and reduced expression of N-cadherin, the key markers of EMT. Interestingly, despite extensive research, the biological function of miR-210 in RCC was not widely investigated. Dias et al. [83] observed a relationship between acute hypoxia, miR-210 excretion, and the increased expression of the chemokine receptor CXCR4, which is related to cancer progression and metastasis. In addition, Wang et al. [88] revealed the simultaneously increased excretion of exosomal miR-210 and decreased expression of vacuole membrane protein VMP1, involved in cancer progression and metastasis, under hypoxic conditions in renal cancer cells. In summary, despite the knowledge discussed, an exact mechanism of action for the particular miRNAs in RCC, and especially the role of their excretion, is not clear thus far. Some authors showed that hypoxia, which is related to rapidly growing tumors, is part of the process by which renal cancer cells excrete such miRNAs [83,88]. In addition, it seems that exosomal miRNAs participate in intracellular communication among tumor-tumor or tumor-endothelial cells [74,88] and possibly disseminate signals for cancer progression. However, it remains largely unknown whether and how exosomal miRNAs contribute to RCC development and progression.

In sum, despite some promising data, no expectations exist that miRNAs will soon be introduced as diagnostic or prognostic biomarkers, neither alone nor in combination with clinical-pathological factors. The comparability and repeatability of current results are disputable, despite the increasing number of miRNA studies. Unstandardized isolation and quantification techniques, as well as the heterogeneity of the study cohorts, and unresolved biological functions are the major hurdles in novel biomarkers research. Thus, the development of standardized methods and functional investigations are urgently needed.

## 4. Biofluid lncRNAs as Biomarkers for Renal Cell Carcinoma

Long noncoding RNAs (lncRNAs) are a class of single RNAs, >200 nucleotides in length, with no protein-coding potential [109]. LncRNAs are involved in gene expression control either by transcriptional regulation through recruiting of chromatin-modifying complexes or by post-transcriptional regulation through interaction with miRNAs, mRNAs, and proteins [110]. In recent years, lncRNAs were shown to contribute to the development of nearly all cancer types, including kidney cancer [111,112]. As in the case of miRNAs, lncRNAs are involved in many processes related to cancer development and progression, including regulation of the cell cycle, proliferation, apoptosis, senescence, migration, invasion, drug resistance, and so on [113,114,115,116]. LncRNAs expression is more tumor- and organ-specific than other RNA entities [117] and they are quite stable in tissue and body fluids such as urine and blood [118]. It is possible that, similar to miRNAs, lncRNAs are protected from RNase degradation by extracellular vesicles and by interactions with specific proteins [119,120]. Thus, lncRNAs may serve as highly specific non-invasive biomarkers and are of particular interest as they may provide more precise diagnostic and prognostic information.

Serum circulating lncRNA from RCC patients was first analyzed by Wu et al. [119]. The authors described five significantly down-regulated lncRNAs in ccRCC patients when compared to healthy controls with an AUC of 5 lncRNAs panel equal to 0.90 and 0.82 for the training and testing sets of samples, respectively (Table 3). Moreover, the panel significantly distinguished ccRCC from benign renal tumors. A similar diagnostic potential was later reported for two single serum-derived lncRNAs, specifically GIHCG and LINC00887, which were investigated by He et al. [121] and Xie et al. [122], respectively. In addition, the investigators observed post-surgical reduction in levels of these lncRNAs in serum, and a higher expression of LINC00887 was related with a shorter OS, which suggested the possibility of using circulating lncRNAs for patient follow-up. Moreover, Qu et al. [110] provided plasma-circulating lncARSR as an independent prognostic factor for RCC patients with sunitinib therapy, by comparing the elevated level of lncARSR in the pre-therapy plasma of RCC patients suffering from progressive disease during sunitinib treatment to patients without progressive disease.

As in the case of miRNAs, the understanding of the biological function of particular lncRNAs is crucial to transfer them to the clinic as a molecular test. The biological basis of lncRNAs in the case of renal cancer was investigated in the three studies [110,121,122]. He et al. [121] and Xie et al. [122] revealed that GIHCG and LINC00887 promoted RCC cells proliferation and migration, and thus may be related to tumor progression; however, the exact molecular pathway remained unclear. The mechanism of action of examined lncRNA was most comprehensively described by Qu et al. [110]. The authors observed that lncARSR served as a sponge, sequestering miR-34 and miR-449, leading to the upregulation of their target receptor tyrosine kinase AXL/c-MET, which in turn activated STAT3, AKT, and ERK signaling pathways, resulting in sunitinib resistance in the RCC cells. Moreover, activated AKT further promoted lncARSR expression by suppressing the transcription factors FOXO1 and FOXO3A, acting as transcription repressors, by recruiting a histone deacetylase. The researches also revealed that lncARSR secretion, from the sunitinib-resistant RCC cells via exosomes, disseminates drug resistance to the sunitinib-sensitive cells. Thus, the results showed that lncARSR may act not only as a clinical biomarker for the monitoring of patients receiving the sunitinib, but also could serve as a therapeutic target to overcome sunitinib resistance in RCC patients.

Despite the described potential, all reported lncRNAs were investigated in a single study, most of which used a small set of samples with an unspecified particular subtype of cancer analyzed (Table 3). However, the specificity of lncRNAs to RCC is seemingly higher when compared to miRNAs or DNA methylation. Thus, lncRNAs appear to have potential as promising, well-performing novel RCC biomarkers.

## 5. Other Epigenetic Phenomena for Non-Invasive Cancer Detection

Another epigenetic phenomenon, such as circulating nucleosomes and their modifications as well as other non-coding RNAs, like P-Element induced wimpy testis (PIWI)-interacting RNAs (piRNAs), may also serve as non-invasive biomarkers for cancers [123,124], while other ncRNAs are far less appropriate as reviewed previously [125].

PiRNA refers to a group of non-coding RNAs, 26–31 nucleotides in length, that maintain genomic stability by silencing transposable elements through CpG methylation, chromatin remodeling, and repression of complementary mRNAs [126]. Recently, it was observed that piRNAs may play an important role in carcinogenesis by driving the inhibition or degradation of oncogenes or tumor suppressor genes, respectively, and the deregulation of various piRNAs was observed in RCC [127,128]. There are two studies on circulating piRNAs in the serum and urine samples of RCC patients [129,130]. Iliev et al. [129] observed a significantly higher level of piR-823 in the serum (*n* = 178) of RCC patients when compared to the healthy controls (*n* = 101), but the diagnostic performance was low with an AUC = 0.63. The better diagnostic potential, with an AUC = 0.74, was established in the urine samples; however, only 20 RCC and 15 healthy control samples were investigated. Meanwhile, Zhao et al. [130] detected downregulated levels of piR-34536 and piR-51810 in ccRCC tissues as compared to normal renal samples, but no significant differences were observed in the serum of ccRCC patients (*n* = 30) in comparison to healthy individuals (*n* = 15). Thus, piRNAs may be promising novel circulating biomarkers of RCC; however, studies on the subject remain quite limited.

Histone modifications mostly include acetylation and methylation of lysine residues, and commonly lead to nearby gene transcriptional activation or repression, respectively, by regulating the access of transcriptional factors to DNA [131]. Deregulation of histone modifications is often involved in tumorigenesis and may also be used as disease biomarkers with the ability to detect such alterations in the biofluids [132,133]. Although utilization of circulating nucleosomes in combination with conventional biomarkers of some cancer types may increase specificity and sensitivity of current tests, as reviewed previously [123], to the best of our knowledge no such investigations were conducted in the field of renal cell carcinoma.

## 6. Conclusions and Future Perspectives

**Figure 1:**
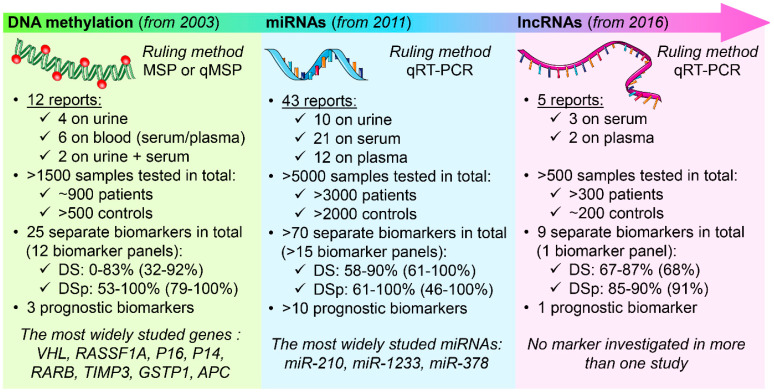
A summary of investigations conducted to date on biofluid circulating epigenetic biomarkers for RCC. This table was created using images from Servier Medical Art Commons Attribution 3.0 Unported License (http://smart.servier.com, accessed on 3 May 2021). Servier Medical Art by Servier is licensed under a Creative Commons Attribution 3.0 Unported License. Abbreviations: DS—diagnostic sensitivity; DSp—diagnostic specificity.

Over the course of almost two decades worth of reports, a considerable number of circulating epigenetic biomarkers of RCC were suggested as possible diagnostic and prognostic tools (Figure 1); however, no marker has reached the clinic yet. The development of a biomarker assay for clinical practice is a multistage process requiring a vast number of samples and validation steps. The majority of the studies conducted thus far, however, lacked internal validation, used relatively small and heterogeneous cohorts, and a minimal number of biomarkers (out of >100 studied) were investigated in more than one study. Most of the studies focused primarily on the diagnostic potential of the particular biomarkers, whereas investigations on the prognostic potential were relatively rare. Moreover, analytical issues, including accuracy, sensitivity, and specificity, were not sufficiently studied and need to be addressed. Thus far, investigators commonly used blood samples (serum/plasma) as a source of nucleic acids, while urine, as a convenient liquid biopsy source for urological cancers, still requires further exploration. Moreover, miRNAs were the most widely studied in terms of potential non-invasive biomarkers for RCC, while, despite their higher stability and earlier occurrence, a limited number of studies focused on DNA methylation. In addition, due to the high specificity and diagnostic potential of lncRNA, further efforts should be made for the wider investigation of these novel biomarkers in the future. Finally, although numerous novel candidate biomarkers were produced, the studies of their biological functions in RCC are scarce; therefore, more detailed insights into their potential mechanism of action in RCC cells are also desirable. Thus, considering renal cancer has the highest mortality rate of all urinary system neoplasms, there is a considerable demand to validate the existing potential biomarkers, and elucidate their biological functions, alongside continuing the search for novel biomarkers with better performance.

Large-scale studies addressing specific DNA methylation, miRNA, or lncRNA patterns in the bodily fluids of patients with RCC are urgently needed for novel biomarker discovery. Next-generation sequencing could be a valuable tool for the rapid screening of liquid biopsy samples in multicenter cohort studies. Moreover, NGS and digital PCR might be more accurate and sensitive than conventional prevailing methods (PCR or qPCR) for the validation of discovered biomarkers. Ultimately, multimarker panels seem to be more informative compared to individual ones and need to be more thoroughly addressed in the future.

## Figures and Tables

**Table 1:** Studies concerning DNA methylation in biofluids as a potential biomarker of renal cell carcinoma.

Reference	Cases	Specimen	Nucleic Acids Extraction Method	Biomarkers Selection Procedure	Method	Biomarker Studied	Methylation Frequency	AUC	DS (%)	DSp (%)	Type of Marker
**Battagli et al., 2003 [24]**	50 cancer cases (35 ccRCC, 6 pRCC, 3 OCT, 2 chRCC, 2 TCC, 1 CDC, and 1 uRCC) + 12 HC	Urine sediments	Phenol/chloroform	From literature	MSP	*VHL*	6/50 (12%)	na	12	100	Dg
						*P16*	4/50 (8%)	na	8	100	
						*P14*	9/50 (18%)	na	18	100	
						*APC*	8/50 (16%)	na	16	100	
						*RASSF1A*	25/50 (50%)	na	50	100	
						*TIMP3*	26/50 (52%)	na	52	100	
						Panel of six biomarkers	45/50 (90%)	na	90	100	
**Hoque et al., 2004 [25]**	26 RCC + 91 controls (various conditions, malignant and non-malignant)	Urine	Phenol/chloroform	From literature	QMSP	*APC*	10/26 (38%)	na	38	96	Dg
						*ARF*	8/26 (31%)	na	31	100	
						*CDH1*	10/26 (38%)	na	38	95	
						*GSTP1*	4/26 (15%)	na	15	100	
						*MGMT*	2/26 (8%)	na	8	100	
						*P16*	9/26 (35%)	na	35	100	
						*RARB2*	8/26 (31%)	na	31	91	
						*RASSF1A*	17/26 (65%)	na	65	89	
						*TIMP3*	12/26 (46%)	na	46	91	
						Panel of nine biomarkers	23/23 (88%)	na	88	na	
**Hoque et al., 2004 [25]**	18 RCC + 30 controls (smokers and non-smokers)	Serum	Phenol/chloroform	From literature	QMSP	*APC*	1/18 (6%)	na	6	97	Dg
						*ARF*	1/18 (6%)	na	6	97	
						*CDH1*	6/18 (33%)	na	33	93	
						*GSTP1*	1/18 (6%)	na	6	100	
						*MGMT*	0/18 (0%)	na	0	97	
						*P16*	4/18 (22%)	na	22	100	
						*RARB2*	1/18 (6%)	na	6	100	
						*RASSF1A*	2/18 (11%)	na	11	97	
						*TIMP3*	3/18 (17 %)	na	17	100	
						Panel of nine biomarkers	12/18 (67%)	na	67	na	
**Urakami et al., 2006 [26]**	33 RCC + 20 HC	Serum	QIAmp DNA Mini Blood kit (Qiagen)	From literature	MSP	*SFRP1*	9/33 (27.3%)	na	27.3	100	Dg
						*SFRP2*	16/33 (48.5%)	na	48.5	100	
						*SFRP4*	8/33 (24.2%)	na	24.2	100	
						*SFRP5*	15/33 (45.5%)	na	45.5	100	
						*DKK3*	9/33 (27.3%)	na	27.3	100	
						*WIF1*	9/33 (27.3%)	na	27.3	100	
Costa et al., 2011 [27] *	50 RCC (34 ccRCC, 7 pRCC, 4 chRCC, and 5 OCT) + 48 HC	Urine sediments	Phenol/chloroform	Gene expression microarrays (Applied Biosystems)	QMSP	*TCF21*	14/50 (52%)	na	28	100	Dg
						*PCDH17*	10/50 (50%)	na	20	100	
						*TCF21 or PCDH17*	16/50 (32%)	0.66	32	100	
**de Martino et al., 2011 [28]**	157 (112 ccRCC, 31 pRCC, and 14 chRCC) + 43 BRT	Serum	QIAamp Ultrasens Virus Kit (Qiagen)	From literature	Restriction endonuclease qPCR	*RASSF1A*	75/200 (37.5%)	0.69	45.9	93.0	Dg
						*VHL*	83/200 (41.5%)	0.71	50.3	90.7	
						*PTGS2*	75/200 (37.5%)	0.52	38.2	65.1	
						*P16*	92/200 (46%)	0.51	46.5	55.8	
**Hauser et al., 2013 [29]**	35 RCC (29 ccRCC, 4 pRCC, and 2 chRCC) + 54 HC	Serum	ChargeSwitch gDNA Kit (Invitrogen)	From literature	Restriction endonuclease qPCR	*APC*	19/35 (54.3%)	0.72	54.3	90.7	Dg
						*GSTP1*	6/35 (17.1%)	0.57	17.1	98.1	
						*P14*	5/35 (14.3%)	0.57	14.3	100	
						*P16*	9/35 (25.7%)	na	25.7	83.3	
						*PTGS2*	8/35 (22.9%)	0.59	22.9	96.3	
						*RARB*	14/35 (40%)	0.61	40.0	85.2	
						*RASSF1A*	8/35 (22.9%)	0.60	22.9	98.2	
						*TIMP3*	20/35 (57.1%)	na	57.1	61.1	
						Panel of eight biomarkers	30/35 (85.7%)	na	85.7	na	
						*APC or GSTP1*	na	0.73	57.1	88.9	
						*APC or PTGS2*	na	0.74	60.0	87.0	
						*APC or PTGS2*	na	0.74	60.0	87.0	
						*APC or RARB*	na	0.76	74.3	77.8	
						*PTGS2 or GSTP1*	na	0.75	62.9	87.0	
**Xin et al., 2016 [30]**	33 RCC + 15 HC	Urine sediments	AllPrep DNA Mini kit (Qiagen)	From literature	Pyrosequencing	*TCF21*	na	na	79	100	Dg
**Skrypkina et al., 2016 [31]**	27 RCC (23 ccRCC, 2 sarcomatoid-like tumors, 1 mixed papillary and ccRCC, and 1 TCC) + 15 HC	Plasma	Proba NA Kit (DNA Technology)	From literature	MSP	*LRRC3B*	20/27 (74.1%)	na	74.1	66.7	Dg
						*VHL*	0/0 (0%)	na	0.0	100	
						*RASSF1*	17/27 (63.0%)	na	63.0	93.3	
						*APC*	14/27 (51.9%)	na	51.9	93.3	
						*FHIT*	15/27 (55.6%)	na	55.6	100	
						*ITGA9*	0/0 (0%)	na	0.0	100	
						*LRRC3B, FHIT, APC* and *RASSF1*	27/27(100%)	na	na	na	
						*RASSF1 or FHIT or APC*	25/27 (92.3%)	na	92.3	86.7	
						*RASSF1 or FHIT*	21/27 (77.8%)	na	77.8	93.3	
						*RASSF1 or APC*	21/27 (77.8%)	na	77.8	93.3	
**Lin et al., 2017 [32]**	142 ccRCC + 34 HC	Serum	QIAmp DNA Blood Mini Kit (Qiagen)	From literature	MSP	*PCDH17*	82/142 (57.7%)	na	57.7	100	Pg
**Jung et al., 2019 [33]**	100 RCC (67 ccRCC + 15 pRCC + 10 chRCC + 8 NA)	Plasma	Dynabeads^®^ SILANE (Thermo Fisher Scientific)	From literature + TCGA	QMSP	*SHOX2*	12/100 (12%)	na	12	>95%	Pg
**Outeiro-Pinho et al., 2020 [34]**	Cohort #1: 53 ccRCC + 57 HC; Cohort #2: 171 ccRCC + 85 HC	Cohort #1: Urine sediments Cohort #2: urine supernatant	Phenol/chloroform	From literature	QMSP	mir-30a	na	0.68/0.67	83/63	53/67	Dg/Pg
**Nuzzo et al., 2020 [35]**	69 RCC (ccRCC, and pRCC) + 13 HC	Plasma	Qiagen Circulating Nucleic Acids Kit (Qiagen)	Illumina HiSeq 4000 (cfMeDIP–seq)	na	Top 300 DMRs	na	0.99	na	na	Dg
**Nuzzo et al., 2020 [35]**	30 RCC + 15 HC	Urine	Genomic DNA Extraction Kit (Qiagen)	Illumina HiSeq 4000 (cfMeDIP–seq)	na	Top 300 DMRs	na	0.86	na	na	Dg

Abbreviations: RCC—renal cell carcinoma; ccRCC—clear cell RCC; pRCC—papillary RCC; chRCC—chromophobe RCC; uRCC—unclassified RCC; HC—healthy control; OCT—oncocytoma; BRT—benign renal tumor; TCC—transitional cell carcinoma; CDC—collecting duct carcinoma; TCGA—the Cancer Genome Atlas; MSP—methylation-specific PCR; QMSP—quantitative MSP; cfMeDIP–seq—cell-free methylated DNA immunoprecipitation and high-throughput sequencing; DMRs—differentially methylated (DNA) regions; AUC—area under the curve; DS—diagnostic sensitivity; DSp—diagnostic specificity; Dg—diagnostic; Pg—prognostic; na—not applicable/available. * The provided AUC, S, and Sp values indicate the prognostic performance for CSS.

**Table 2:** Studies concerning microRNAs in biofluids as potential biomarkers of renal cell carcinoma.

Reference	Sample Size	Sample	RNA Isolation	Biomarker Selection	Method (Reference)	Biomarker Studied, Regulation	AUC	DS (%)	DSp (%)	Type of Marker
Wulfken et al., 2011 [58] **	Screening phase: 6 ccRCC + 6 HC; Validation phase#1: 33 ccRCC + 30 HC; Validation phase#2: 84 RCC (69 ccRCC, 10 pRCC, 3 chRCC, and 2 sRCC) + 106 controls (93 HC, 3 AML, and 10 OCT)	Serum	mirVana PARIS Kit (Ambion)	TLDA (tissue and serum)	qRT-PCR (TaqMan) (miR-39)	miR-1233 ↑	0.59	77.4	37.6	Dg
**Zhai et al., 2012 [59]**	10 RCC + 10 HC	Plasma	TRIzol (Invitrogen)	Sequencing (on tissue-derived RNA)	qRT-PCR (Qiagen) (RNU6)	miR-508-3p ↓	na	na	na	na
**Brandenstein et al., 2012 [60]**	10 RCC + 35 controls (5 OCT, 1 AML, 9 RCC regressive, and 15 various inflammation or malignancies)	Whole urine	miRNeasy kit (Qiagen)	From literature	qRT-PCR (TaqMan) (5S rRNA)	miR-15a ↑	na	na	na	na
**Redova et al., 2012 [61]**	Exploratory phase: 15 ccRCC + 12 HC; Validation phase: 90 RCC (73 ccRCC, 8 pRCC, and 9 chRCC) + 35 HC	Serum	miRNeasy Mini Kit (Qiagen)	TLDA	qRT-PCR (TaqMan) (miR-16)	miR-378 ↑	0.71	70.0	60.0	Dg
						miR-451 ↓	0.77	81.0	77.0	
						miR-378 and miR-451	0.86	81.0	83.0	
Hauser et al., 2012 [62] **	Discovery cohort: 25 ccRCC + 25 cancer-free controls; Validation cohort: 117 RCC (104 ccRCC, 10 pRCC, 1 chRCC, 1 sRCC) + 123 CTRL (109 cancer-free, 14 BRT)	Serum	mirVana PARIS Kit (Applied Biosystem)	From Wulfken et al., 2011	qRT-PCR (TaqMan) (miR-39)	miR-378 ↑	0.73	na	na	na
**Zhao et al., 2013 [63]**	68 ccRCC + 42 HC	Serum	MicroMini Kit (Qiagen)	From literature	qRT-PCR (Qiagen) (5S rRNA)	miR-210 ↑	0.87	81.0	79.4	Dg
**Cheng et al., 2013 [64]**	12 ccRCC + 12 BKL	Serum	mirVana™ PARIS kit (Applied Biosystems)	From literature	qRT-PCR (Takara) (RNU6)	miR-34a ↑	na	na	na	Dg
						miR-21 ↑	na	na	na	
						miR-224 ↑	na	na	na	
						miR-141 ↓	na	na	na	
**Zhao et al., 2013 [65]**	30 ccRCC + 50 HC	Plasma	TRIzol (Invitrogen)	From literature	qRT-PCR (Takara)	miR-187 ↓	na	na	na	na
**Iwamato et al., 2014 [66]**	34 ccRCC + 23 HC	Serum	microRNA extractor SP kit (Wako)	From literature	qRT-PCR (TaqMan) (miR-16)	miR-210 ↑	0.77	65	83	Dg
**Teixeira et al., 2014 [67]**	43 RCC (31 ccRCC + 12 others) + 34 HC	Plasma	mirVana™PARIS™ Kit (Ambion^®^)	From literature	qRT-PCR (TaqMan) (RNU44)	miR-221 ↑	0.70	72.5	33.3	Dg/Pg
						miR-222 ↑	na	na	na	
**Wang et al., 2015 [68]**	Screening phase: 25 ccRCC + 25 controls (ns); Validation phase: 107 ccRCC (randomly divided into two sets: 28 + 79) + 107 controls (ns)	Serum	Phenol/chloroform	TLDA	qRT-PCR (TaqMan) (let-7d/g/i)	miR-193a-3p ↑	na	na	na	Dg
						miR-362 ↑	na	na	na	
						miR-572 ↑	na	na	na	
						miR-28-5p ↓	na	na	na	
						miR-378 ↓	na	na	na	
						Panel of all 5 miRNAs	0.80	80.0	71.0	
**Zhang et al., 2015 [69]**	82 RCC (ns) + 19 HC	Serum	TRIzol (Invitrogen)	From literature	qRT-PCR (Takara) (RNU6)	miR-183 ↑	na	na	na	na
Fedorko et al., 2015 [70] **	195 RCC (157 ccRCC, 26 pRCC, and 12 chRCC) + 100 HC	Serum	miRNeasy Mini Kit (Qiagen)	From literature	qRT-PCR (TaqMan)	miR-378 ↑	0.82	na	na	Dg/Pg
						miR-210 ↑	0.74	na	na	
						miR-378 and miR-210	0.85	80.0	78.0	
**Liu et al., 2015 [71]**	32 RCC (ns) + 32 HC	Serum	TRIzol (Invitrogen)	From literature	qRT-PCR (BulgeLoop) (RNU6)	miR-210 ↑	na	na	na	na
Zhang et al., 2016 [72] ***	82 RCC + 80 HC	Serum	MicroMini kit (Qiagen)	From literature	qRT-PCR (Qiagen) (RNU6)	miR-210 ↑	0.69	70.0	62.2	Dg
						miR-1233 ↑	0.82	81.0	76.0	
**Tusong et al., 2016 [73]**	30 ccRCC + 30 HC	Serum	mirVana PARIS Kit (Ambion)	From literature	qRT-PCR (Maxima) (RNU6)	miR-21 ↑	0.87	77.3	96.4	Dg
						miR-106a ↑	0.82	86.7	70.0	
Butz et al., 2016 [74] ***	Discovery cohort: 28 ccRCC + 18 HC; Validation cohort: 105 (81 ccRCC, 24 BRT) + 33 HC	Urine sediments	miRNeasy Serum/Plasma Kit (Qiagen)	Screening of 754 miRNA by qRT-PCR (TaqMan)	qRT-PCR (TaqMan) (miR-16-5p-miR-106a-5p)	miR-126-3p and miR-34b-5p ↓	0.79	77.5	72.4	Dg
						miR-126-3p and miR-449a ↑	0.84	83.8	62.5	
						miR-150-5p/miR-126-3p ↓	0.77	72.5	75.9	
						miR-126-3p and miR-486-5p	0.85	75.0	87.5	
**Li at al, 2017 [75]**	75 ccRCC + 45 HC	Urine supernatant	Micro Mini Kit (Qiagen)	From literature	qRT-PCR (Qiagen) (cel-miR-39)	miR-210 ↑	0.76	57.8	80.0	Dg
**Yadav et al., 2017 [76]**	30 ccRCC + 15 controls with non-renal benign diseases (urethral stricture or benign prostatic enlargement)	Serum	miRNA Serum/Plasma kit (Qiagen)	From literature	qRT-PCR (Qiagen) (cel-miR-39)	miR-34a ↓	0.92	80.7	80.0	Dg
						miR-141 ↓	0.78	75.0	73.3	
						miR-1233 ↑	0.97	93.3	100	
						miR-141 and miR-1233	na	100	73.3	
						miR-1233 and miR-34a	na	96.6	80.0	
						miR-141 and miR-34a	na	73.3	60.0	
						miR-34a, miR-141, and miR-1233	na	100	60.0	
Chanudet et al., 2017 [77] **	94 ccRCC + 100 controls (ns)	Plasma	NucleoSpin^®^ miRNA Plasma kit (Macherey-Nagel).	TaqMan arrays (A + B cards) (RNU6 and let-7g/d/i)	na	miR-150 ↓	na	na	na	Pg
						miR-451 ↓	0.64	na	na	
						miR-451 and miR-26b	0.66	na	na	
Du et al., 2017 [78] ***	Screening cohort: 44 RCC (40 ccRCC + 2 pRCC + 2 uRCC); Validation cohort: 65 RCC (52 ccRCC, 6 pRCC, 2 chRCC, and 5 uRCC)	Plasma	miRNeasy Micro Kit (Qiagen)	RNA sequencing (Illumina HiSeq2000)	qRT-PCR (TaqMan) (miR-127-3p)	miR-let-7i-5p	na	na	na	Pg
						hsa-miR-190b	na	na	na	
						hsa-miR-26a-1-3p	na	na	na	
						hsa-miR-145-3p	na	na	na	
						hsa-miR-200a-3p	na	na	na	
						hsa-miR-9-5p	na	na	na	
						hsa-miR-615-3p	na	na	na	
**Lou et al., 2017 [79]**	Discovery cohort: 10 (ccRCC 5 preoperative and 7 days after operation); Validation cohort: 153 (106 ccRCC + 19 ncRCC + 28 AML) + 123 HC	Plasma	TRIReagent BD (Molecular Research)	miRNA microarray (Agilent)	qRT-PCR (Thermo) (RNU6B, cel-miR-39, miR-320c)	miR-144-3p ↑	0.91	87.1	83	Dg/Pg
**Petrozza et al., 2017 [80]**	38 ccRCC + 10 HC from two independent cohorts	Whole urine	miRNAeasy serum/plasma kit (Qiagen)	From previous study by the same group	qRT-PCR (Qiagen) (Spike-In Control (Qiagen))	miR-210-3p ↑	na	na	na	na
**Fedorko et al., 2017 [81]**	69 ccRCC + 36 HC (surgically treated for various benign urological conditions)	Urine supernatant	Urine microRNA Purification Kit (Norgen Biotek)	From literature	qRT-PCR (TaqMan) (syntetic miRNA oligo (IDT))	let-7a	0.83	71.0	81.0	Dg
						let-7b	0.75	73.0	67.0	
						let-7c	0.67	65.0	62.0	
						let-7d	0.66	66.0	61.0	
						let-7e	0.65	62.0	61.0	
						let-7g	0.69	70.0	60.0	
						Panel of all 6 miRNAs	0.83	na	na	
**Wang et al., 2017 [82]**	27 RCC (ns) + 28 controls	Serum	na	From literature	qRT-PCR (Sangon Biotech) (GAPDH)	miR-429 ↑	na	na	na	Dg/Pg
**Dias et al., 2017 [83]**	54 RCC (39 ccRCC + 15 other) + 50 HC	Plasma	GRS microRNA kit (Grisp^®^)	From literature	qRT-PCR (TaqMan) (RNU48)	* miR-210 ↑	0.70	60.9	73.1	Pg
						* miR-218 ↑	na	na	na	
						* miR-221 ↑	0.62	71.4	65.0	
						* miR-1233 ↑	0.61	39.1	92.6	
Li et al., 2017 [84] **	139 RCC + 139 HC	Serum	TRIzol (Invitrogen)	From literature	qRT-PCR (Takara) (RNU6)	miR-22 ↓	na	na	na	Pg
**Heinemann et al., 2018 [85]**	Discovery cohort: 18 ccRCC + 8 BRT (4 OCT and 4 complicated renal cysts); Validation cohort: 115 (68 ccRCC, 17 OCT, 14 AML, and 16 complicated renal cysts) + 28 HC	Serum	mirVana PARIS Kit(Thermo Fisher Scientific)	Sequencing (Illumina NextSeq 500)	qRT-PCR (Qiagen) (miR-16, miR-191-5p, miR-320a)	miR-122-5p ↓	0.71	na	na	Pg
						miR-206 ↓	0.73	83.8	57.1	
						miR-122-5p and miR-206	0.73	na	na	
Mytsyk et al., 2018 [86] **	52 RCC (22 ccRCC, 16 pRCC, and 14 chRCC) + 15 BRL (8 OCT, 2 PA, and 5 AML) + 15 HC	Whole urine	mirVana miRNA Isolation Kit (Applied Biosystems)	From literature	qRT-PCR (TaqMan) (RNU6)	miR-15a ↑	0.96	98.0	100	Dg
**Chen et al., 2018 [87]**	66 ccRCC + 67 HC	Plasma	TRIzol (Thermo Fisher Scientific)	From previous study by the same group	qRT-PCR (Invitrogen) (cel-miR-39)	miR-210 ↑	0.68	89.6	48.5	Dg
						miR-224 ↑	0.61	88.1	40.9	
						miR210 and miR-224	0.66	92.5	45.5	
Wang et al., 2018 [88] ***	Discovery cohort: 5 ccRCC + paired NRT tissue; Validation cohort: 45 RCC (ns) + 30 HC	Serum	TRIzol(Invitrogen)	miRNA Microarray (Agilent Technologie)	qRT-PCR (Invitrogen) (miR-16)	miR-210 ↑	0.79	67.5	70.0	Dg
						miR-210 ↑ (Exo)	0.88	82.5	80.0	
Song et al., 2019 [89] ***	70 ccRCC + 30 HC	Urine sediments	TRIzol Plus RNA Purification Kit (Life Technologies)	Sequencing (Illumina HiSeq 2000)	qRT-PCR (TaqMan)	miR-30c-5p ↓	0.82	68.6	100	Dg
**Liu et al., 2019 [90]**	Testing stage: 10 ccRCC + 10 HC; Validation stage: 85 ccRCC + 35 HC	Serum	TRI zol^®^ LS (Invitrogen)	GEO + TCGA	qRT-PCR (GenePharma) (miR-39)	miR-508-3p ↓	0.80	na	na	Dg
						miR-885-5p ↑	0.87	na	na	
						miR-508-3p and miR-885-5p	0.90	na	na	
**Zhao et al., 2019 [91]**	50 ccRCC + 74 HC	Serum	miRNeasy Serum/Plasma Kit (Qiagen)	TCGA	qRT-PCR (Qiagen) (cel-miR-54)	miR-625-3p ↓	0.79	70.3	80.0	Dg
**Petrozza et al., 2019 [92]**	21 ccRCC + 16 HC	Whole urine	miRNeasy Serum/Plasma Kit (Qiagen)	From previous study by the same group	qRT-PCR (Qiagen) (C. elegans miR-39)	miR-210-3p ↑	na	na	na	na
**Di Meo et al., 2020 [93]**	Discovery cohort: 9 SRM (6 ccRCC + 3 OCT) Validation cohort: 71 SRM (44 ccRCC + 27 OCT)	Urine	miRNeasy Serum/Plasma Kit (Qiagen)	Screening of 754 miRNA by qRT-PCR (TaqMan)	qRT-PCR (TaqMan) (Geometric mean of miR-204, miR-1825, RNU48, and RNU6)	has-miR-432-5p ↑	0.71	na	na	Pg
						has-miR-532-5p ↑	0.70	na	na	
						has-miR-10a-5p ↑	0.66	na	na	
						has-miR-144-3p ↑	0.68	na	na	
						has-miR-28-3p ↑	0.65	na	na	
						has-miR-326 ↑	0.68	na	na	
						has-miR-328-3p ↑	0.65	na	na	
						has-miR-603 ↑	0.67	na	na	
						has-miR-93-3p ↑	0.68	na	na	
**Xiao et al., 2020 [94]**	Discovery cohort: 5 ccRCC (preoperative and 7 days after surgery); Validation cohort: 18 ccRCC (preoperative and 7 days after surgery)	Plasma	Trizol (Thermo)	miRNA Microarray (Agilent Technologies)	qRT-PCR (Thermo) (RNU6)	miR-765	na	na	na	na
Kalogiroum et al., 2020 [95] **	67 pRCC (34 pRCC type 1, 33 pRCC type 2) + 33 controls	Serum	Exiqon RNA services (http://www.exiqon.com, accessed on 1 August 2021)	From publications, TCGA	qRT-PCR (Exiqon) (miR-23a-3p, miR-191-5p, and miR-103a-3p)	miR-21-5p ↑	0.57	na	na	na
						miR-210-3p ↓	0.71	na	na	
						miR-21-5p and miR-210-3p	0.72	na	na	
Dias et al., 2020 [96] ***	Group A: 32 ccRCC (localized); Group B: 37 ccRCC (metastatic)	Plasma	Plasma/Serum RNAPurification Mini Kit (Norgen Biotek Corporation)	From publications (related to hypoxia)	qRT-PCR (TaqMan) (hsa-let7a-5p, hsa-miR-16-5p)	hsa-miR-25-3p	na	na	na	Pg
						hsa-miR-126-5p	na	na	na	
						hsa-miR-200c-3p	na	na	na	
						hsa-miR-301a-3p	na	na	na	
						hsa-miR-1293	na	na	na	
**Wang et al., 2020 [97]**	12 ccRCC (preoperative and postoperative)	Plasma	na	GEO	qRT-PCR (na) (RNU6)	miR-483-5p	na	na	na	na
**Huang et al., 2020 [98]**	Screening stage: 20 RCC (ns) + 20 HC; Testing stage: 30 RCC (ns) + 30 HC; Validation stage: 76 RCC (ns) + 80 HC	Serum	TRIzol LS(Invitrogen)	From publications	qRT-PCR (Takara) (cel-miR-39)	miR-224-5p ↑	0.69	na	na	Pg
						miR-34b-3p ↓	0.78	na	na	
						miR-129-2-3p ↓	0.69	na	na	
						miR-182-5p ↓	0.75	na	na	
						miR-224-5p, miR-34b-3p, and miR-182-5p	0.86	80.3	66.3	
Xiao et al., 2020 [99] ***	Discovery cohort: 5 RCC + 5 controls; Validation cohort: 22 RCC (18 ccRCC + 4 pRCC) + 16 HC	Plasma	miRNeasy kit (Qiagen)	Sequencing (Illumina NovaSeq 6000)	qRT-PCR (na) (miR-16-5p)	hsa-miR -92a-1-5p ↓	0.83	87.5	77.3	Dg
						hsa-miR-424-3p ↑	0.77	75.0	81.8	
						hsa-miR-149-3p ↑	0.72	75.0	73.0	
**Cochetti et al., 2020 [100]**	13 ccRCC + 14 HC	Whole urine	miRNeasy Micro Kit (Qiagen)	GEO	qRT-PCR (Qiagen) (miR-16, cel-miR-39, and miRTC)	miR-122 ↑	0.82	na	na	Dg
						miR-1271 ↑	0.79	na	na	
						miR-15b	0.59	na	na	
						miR-122, miR-1271, and miR-15b (7p-urinary score)	0.96	100	86	

Abbreviations: RCC—renal cell carcinoma; ccRCC—clear cell renal cell carcinoma; pRCC—papillary RCC; chRCC—chromophobe RCC; sRCC—sarcomatoid RCC; uRCC—unclassified RCC; ncRCC—non-clear cell RCC; HC—healthy control; AML—angiomyolipoma; OCT—oncocytoma; PA—papillary adenoma; BRT—benign renal tumor; BKL—benign kidney lesions; SRM—small renal masses; TLDA—TaqMan Low-Density Arrays; TCGA—The Cancer Genome Atlas; GEO—Gene Expression Omnibus; qRT-PCR—quantitative real-time PCR; AUC—area under the curve; DS—diagnostic sensitivity; DSp—diagnostic specificity; Dg—diagnostic; Pg—prognostic; na—not applicable/available; ns—not specified. ↑/↓—upregulated/downregulated level of miRNAs; ** Multicenter studies; *** Authors investigated exosomal or microvesicles derived miRNA.

**Table 3:** Studies concerning lncRNAs in biofluids as a potential biomarkers of renal cell carcinoma.

Reference	Sample Size	Sample	RNA Isolation	Biomarker Selection	Method (Reference)	Biomarker Studied, Regulation	AUC	DS (%)	DSp (%)	Type of Marker
**Wu et al., 2016 [119]**	Training set: 24 ccRCC + 27 HC Testing set#1: 37 ccRCC + 35 HC; Testing set#2: 10 ccRCC + 8 BRT	Serum	Blood Total RNAIsolation Kit (BioTeke)	lncRNA Database (82 lncRNA related to the cancer)	qRT-PCR (Takara) (β-actin)	lncRNA-LET ↓	na	na	na	Dg
						PVT1 ↓	na	na	na	
						PANDAR ↓	na	na	na	
						PTEMP1 ↓	na	na	na	
						LINC00963 ↓	na	na	na	
						Panel of 5 lncRNA	0.90/0.82	79.2/67.6	88.9/91.4	
**Qu et al., 2016 [110]**	71 RCC (ns)	Plasma	mirVana PARIS Kit (Ambion)	lncRNA + mRNAmicroarrays (Agilent)	qRT-PCR (Takara) (β-actin)	lncARSR	na	na	na	Pg
**He et al., 2018 [121]**	46 RCC (ns) + 46 HC	Serum	TRIzol Reagent (Invitrogen)	From literature	qRT-PCR (Takara) (β-actin)	GIHCG ↑	0.92	87.0	84.8	Dg
**Xie et al., 2020 [122]**	114 RCC (ns) + 79 HC	Serum	TRIzol LS (Invitrogen)	From GEPIA database	qRT-PCR (Qiagen) (Cel-miR-39)	LINC00887 ↑	0.80	67.1	89.9	Dg/Pg
**Zhang et al., 2020 [118]**	Discovery set: 5 ccRCC + 5 HC; Validation set: 24 ccRCC	Plasma	TRIzol (Invitrogen)	Arraystar lncRNA microarrays (KangChen Biotech)	qRT-PCR (Nuoweizan Biotech) (β-actin)	SOCS2-AS1 ↓	na	na	na	na

Abbreviations: RCC—renal cell carcinoma; ccRCC—clear cell renal cell carcinoma; HC—healthy control; BRT—benign renal tumor; GEPIA—gene expression profiling interactive analysis; qRT-PCR—quantitative real time PCR; AUC—area under the curve; DS—diagnostic sensitivity; DSp—diagnostic specificity; Dg—diagnostic; Pg—prognostic; na—not applicable/available; ns—not specified. 6. Conclusions and future perspectives. ↑/↓—upregulated/downregulated level of lncRNAs.

## Data Availability

Not applicable.
